# The Effect of Education through Short Message Service (SMS) Messages on Diabetic Patients Adherence

**DOI:** 10.3390/scipharm85020023

**Published:** 2017-05-12

**Authors:** Wirawan Adikusuma, Nurul Qiyaam

**Affiliations:** Faculty of Health Science, University of Muhammadiyah Mataram, Mataram 83127, Indonesia; nuqi.gra@gmail.com

**Keywords:** Short Message Service (SMS), adherence, glycemic control, diabetes

## Abstract

Poor adherence and a lack of understanding of medication instructions for oral antidiabetic use are key factors that inhibit the control of glycemic levels. The aforementioned situation needs intervention to improve medication adherence and the therapy. This study was conducted with a quasi-experimental design with prospective data collection. The subjects of this study were 50 outpatients with type 2 diabetes melitus (T2DM) who had received oral antidiabetic medicine therapy at least six months prior to adherence measurement. The patients were classified into two groups—the control group and the intervention group. The intervention group received Short Message Service (SMS) messages of diabetes education, while the control group did not. Data collection was conducted by doing interviews and administering the Morisky Medication Adherence Scale (MMAS) questionnaire. The results showed the increase in adherence in the intervention group as 1.15 ± 1.04 and that in the control group as 0.72 ± 0.90. These results indicated that there were significant differences in MMAS score between the control and intervention groups (*p* < 0.05). The decrease in fasting blood glucose and glucose measured 2 h postprandially was greater in the intervention group than that in the control group. It was concluded that the provision of education through SMS had a positive effect on medication adherence and glycemic levels.

## 1. Introduction

Type 2 diabetes mellitus (T2DM) is a metabolic disorder with symptoms of chronic hyperglycemia as a result of insulin resistance or a deficiency of insulin secretion. Uncontrolled glucose levels can cause acute and chronic complications that can lead to dysfunction and failure of various organs [1]. Barriers to optimal health outcomes in T2DM can be attributed to low patient adherence to anti-diabetic medication as well as a lack of understanding of the recommended medication regimen [2]. Lifestyle modification and adherence to antidiabetes medications are the major determinants of therapeutic success in the management of diabetes [3]. In addition, other studies have found a significant association between the adherence of antidiabetes medications and glycemic control [4]. In one study, pharmacists were used to improve patient understanding of anti-diabetic medications and were found to improve patients’ medication adherence.

Medication adherence can be improved using Short Message Service (SMS) or “text” reminders, voice reminders, and special applications (“apps”) to remind patients to take medications. Specifically, SMS or text reminders are minimally intrusive to patients’ privacy and can be delivered through simpler mobile phones, enabling potential access to a larger number of clients. Hence, SMS reminders offer a promising method of promoting the medication adherence of patients, especially those who suffer from chronic conditions and are required to take medications for a long period of time [5].

SMS, in conjunction with a behavior modification component, has been shown to reduce T2DM risk factors in patients involved in smoking cessation [6]. Additionally, SMS is a low-cost intervention that can be a vital to communicating the importance of medication adherence to patients [7]. Furthermore, a study using Real Time Medication Monitoring (RTMM) with SMS can improve patient adherence with T2DM once a treatment regimen has been established [8]. This study aimed to determine the effect of SMS toward adherence and glycemic levels of people with T2DM.

## 2. Methods

**Figure 1:**
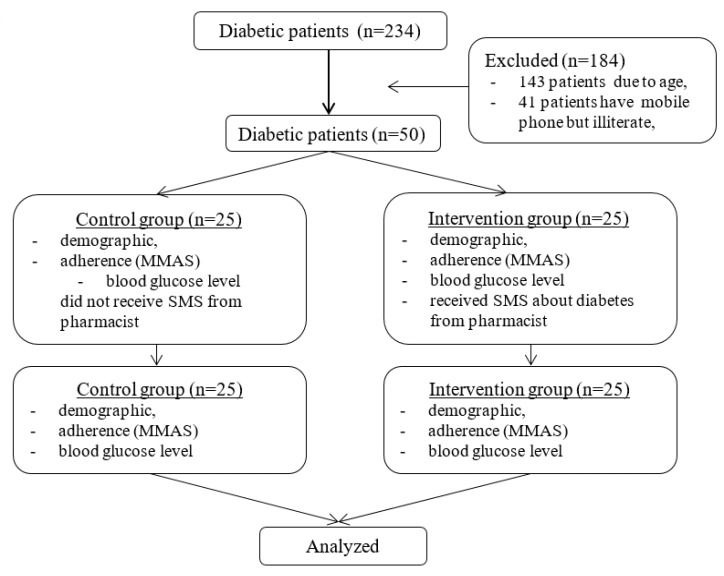
Flowchart of study participants. MMAS, Morisky Medication Adherence Scale.

This study was conducted by a quasi-experimental design with prospective data collection. The research subjects were outpatients with T2DM at a hospital in Mataram, Indonesia, who had received oral antidiabetic medicine therapy at least six months prior to adherence measurement. Subjects who meet the inclusion criteria were 50 patients with T2DM. Inclusion criteria required subjects to have T2DM, have been taking oral antidiabetic medication for at least six months, be aged 45–65 years old, have access to a mobile phone, and be able to read SMS on a mobile phone. Exclusion criteria rejected those who were deaf, pregnant, or unwilling to participate in this research. The selection of subjects for each group was performed randomly by a research assistant. Subjects who met the inclusion criteria were divided into two groups: the control group and the treatment group (Figure 1). All participants in the intervention group received an SMS message randomly once a day until their second visit (post-study). All patients potentially received the same SMS content, while the control group did not receive any SMS messages. We ensured that all participants in the study could read the SMS messages. Some examples of SMS messages are provided in Table 1.

The data collection was conducted by doing interviews and administering Morisky Medication Adherence Scale (MMAS) questionnaires. MMAS consists of eight questions and the level of adherence is measured from the range of 0–8, and categorized into three levels of medication adherence: high adherence (score 8), moderate adherence (score 6 – <8), and low adherence (score < 6). This study was approved by the ethics committee of West Nusa Tenggara Provincial Hospital, and written informed consent was obtained from all study subjects.

## 3. Results

There were 50 patients with T2DM who met the inclusion criteria. Table 2 shows the characteristics of the study subjects. Most of the study subjects were female (56%), had an education level up to senior high school (84%), did not have a job (80%), had an average treatment duration of less than 5 years (80%), was aged ≥55 years (72%), and took medicine as part of a combination therapy (60%). The average of glucose measured 2 h postprandially was 258.1 ± 108.9 in the control group and 234.4 ± 84.5 in the intervention group, whereas the average fasting glucose was 195.68 ± 92.76 in the control group and 162.6 ± 63.5 in the intervention group.

Table 3 shows the adherence of patients with T2DM between the two groups. The analysis shows the increased adherence in the control group (0.72 ± 0.902) and the treatment group (1.15 ± 1.043). These results indicate that there are significant differences between the MMAS scores in the control and treatment groups (*p* < 0.05).

Table 4 shows the results of chi square analysis between the characteristics of the study subjects with T2DM adherence. Characteristic factors include gender, education, occupation, treatment duration, and age. None of them affect adherence statistically (*p* > 0.05). These results are similar to results of research conducted by Puspitasari et al. (2012), where none of the subjects affected adherence [9]. Poor adherence in the use of the medicine can occur in men and women of all ages, at all levels of education, and at all economic levels [10].

Table 5 and Table 6 show the measurement of blood glucose before and after treatment. Fasting blood glucose and blood glucose measured 2 h postprandially, before and after treatment, are two of the therapeutic efficacy parameters measured in this study. Based on the results of these measurements, a decrease in the fasting blood glucose in the control group (19.88 ± 45.56) and treatment group (25.6 ± 52.19) and a decrease in blood glucose measured 2 h postprandially in the control group (19.88 ± 55.88) and the treatment group (27.36 ± 80.16) can be seen. These results indicate that there is no difference between fasting blood glucose levels and glucose levels measured 2 h postprandially in the control group or in the treatment group (*p* > 0.05).

## 4. Discussions

Table 2 shows the baseline data of MMAS and glycemic levels for the control and intervention groups. The baseline data research is needed to determine whether the samples of the control group and the treatment group before receiving SMS intervention of pharmacists have similarities or differences. Based on the analysis using an independent *t*-test, it can be seen that there was no significant difference between the two groups. The effect of the intervention of SMS on the treatment group can be clearly seen.

The results of this study showed that the administration of education in the form of SMS messages from pharmacists can significantly improve patient adherence (*p* < 0.05). This is because intervention in the form of SMS messages can improve T2DM patients’ understanding of treatment. This is supported by previous studies that stated that the use of SMS messaging can significantly improve patient medication adherence on schedule [11]. Patients are more likely to take medicine when they realize that T2DM is a serious disease with serious consequences [12]. Adherence to therapy is an important component of any treatment regimen, and pharmacists are ideally positioned to influence a patient’s medication adherence in a positive way [13]. A mobile phone SMS has the potential to communicate with diabetes patients and to build an awareness of the disease, improve self-management, and avoid complications [14].

The success of medical treatment is determined by the quality of a patient’s health services and their adherence to their treatment plan [15]. The success of the therapy in this study is evidenced by the decrease in fasting blood glucose and glucose measured 2 h postprandially. The decline in fasting blood glucose and glucose measured 2 h postprandially in the control group compared to the treatment groups did not differ significantly (*p* > 0.05). Although statistically there was no difference, clinically, a decline in fasting blood glucose and glucose measured 2 h postprandial provided greater improvements in the treatment group who received a text message of T2DM education compared with the control group. This can be due to education through diabetes-related SMS given to the intervention group being able to improve patient knowledge to participate positively in the treatment.

Adherence plays an important role in achieving the success of therapy, especially for chronic diseases such as diabetes mellitus [16]. Therapeutic treatments will not achieve optimum effects without patient awareness; without it, therapy can fail, or complications may eventually lead to fatal events [17]. Low patient adherence to the treatment of diabetes mellitus is one of the causes of low blood glucose level control. With additional education by pharmacists in the form of SMS messages, the workload of pharmacists in terms of convincing and reminding patients that the medicine prescribed is useful for improving their quality of life is expected to decline. Indeed, the greatest challenge for pharmacists, in the effort to help patients cope with illness, is increasing patient awareness of the importance of adherence.

## 5. Conclusions

The provision of education through SMS had a positive effect on medication adherence and glycemic levels.

## Figures and Tables

**Table 1:** Content of SMS.

No	Short Message Service
1.	Good morning, regular physical exercise or walking habit will help to bring your blood glucose to normal level. Get well soon, thank you.
2.	Good morning, keep your diet and keep exercise. Get well soon, thank you.
3.	Good morning, taking medication timely will help to keep your blood sugar in control and avoid complications. Get well soon, thank you.
4.	Good morning, a healthy diet will keep you healthy and happy. Get well soon, thank you.
5.	Good morning, if you are stressed, stop your work. Go for walk and relax. Get well soon. Thank you
6.	Good morning, avoid alcoholic beverages alcoholic beverages can affect your blood sugar levels. Get well soon, thank you.
7.	Good morning, avoid or limit your consumption of simple or refined sugary foods, as they can quickly transform into glucose as they enter your body. Get well soon, thank you.
8.	Good morning, starchy foods such as corn, peas, potatoes, white rice and white pasta are high in carbohydrates, which can convert quickly into sugar and negatively affect your glucose levels. Get well soon, thank you.
9.	Good morning, consumption protein from vegetable sources, low fat milk products, fish and lean meat is preferable. Get well soon, thank you.
10.	Good morning, whole fruits are recommended in moderation (1–2 servings) however, very sweet fruits should be avoided. Get well soon, thank you.

**Table 2:** Subject’s characteristics and baseline data for the control and intervention groups.

Characteristics		N = 50		(*p*-Value)
		Control (n = 25)	Intervention (n = 25)	
**Gender**	Male	11	14	0.331
	Female	14	11	
**Education Level**	Up to Senior high school	20	22	0.829
	Undergraduate	5	3	
**Occupation**	Working	6	4	0.176
	Jobless	19	21	
**Treatment Duration**	<5 years	10	11	0.707
	≥5 years	15	14	
**Age**	<55 years	4	10	0.909
	≥55 years	21	15	
**Treatment**	Monotherapy	10	10	0.668
	Combination	15	15	
**Glucose, measured 2 h postprandially**		258.1 ± 108.9	234.4 ± 84.5	0.493
**Fasting blood glucose**		195.68 ± 92.76	162.6 ± 63.5	0.410

*p*-value of independent *t*-test.

**Table 3:** MMAS scores in the control and intervention groups (Mean ± SD).

Group	Mean ± SD	*p*-Value a	Δ	*p*-Value b
Pre control	6.84 ± 1.20	0.001 *	0.72 ± 0.90	0.019 *
Post control	7.56 ± 0.63			
Pre intervention	6.74 ± 1.20	0.000 *	1.15 ± 1.04	
Post intervention	7.89 ± 0.26			

* *p* < 0.05. *p*-value a: *p*-value of Wilcoxon, *p*-value b: *p*-value of Mann–Whitney, Δ: Increase score MMAS.

**Table 4:** Chi square analysis between the characteristics of the research subject to adherence.

Characteristics	Adherence		Asymp. Sig.	RR *for Cohort*	95% C. I	
	8 High	6 – <8 Moderate			Lower	Upper
Gender						
Male	10	18	0.05	0.561	0.311	1.011
Female	14	8				
Education Level						
Up to senior high school	20	22	0.902	0.952	0.444	2.041
Undergraduate	4	4				
Occupation						
Working	19	23	0.370	0.724	0.385	1.361
Jobless	5	3				
Treatment Duration						
<5 years	11	10	0.598	1.168	0.658	2.074
≥5 years	13	16				
Age						
<55 years	9	5	0.151	1.543	0.891	2.673
≥55 years	15	21				
Treatment						
Monotherapy	10	10	0.817	1.071	0.599	1.917
Combination therapy	14	16				

**Table 5:** Glucose measured 2 h postprandially.

Group	Mean ± SD	*p*-Value a	Δ	*p*-Value b
Pre control	247.36 ± 95.86	0.088	19.88 ± 55.88	0.566
Post control	227.48 ± 82.00			
Pre intervention	268.76 ± 121.62	0.101	27.36 ± 80.16	
Post intervention	241.40 ± 88.10			

* *p* < 0.05. *p*-value a: *p*-value of paired *t*-test, *p*-value b: *p*-value of independent *t*-test, Δ: decrease in glucose measured 2 h postprandially.

**Table 6:** Fasting blood glucose.

Group	Mean ± SD	*p*-Value a	Δ	*p*-Value b
Pre control	175.12 ± 81.63	0.039 *	19.88 ± 45.56	0.414
Post control	155.24 ± 60.10			
Pre intervention	195.68 ± 92.76	0.022 *	25.6 ± 52.19	
Post intervention	170.08 ± 67.14			

* *p* < 0.05. *p*-value a: *p*-value of paired *t* test, *p*-value b: *p*-value of independent *t* test, Δ: decrease in fasting blood glucose.
